# A Hidden Coordination-Bond Torsional Deformation as a Sign of Possible Spin Transition in Nickel(II)-Bis(nitroxide) Compounds

**DOI:** 10.3390/molecules25173790

**Published:** 2020-08-20

**Authors:** Yukiya Kyoden, Takayuki Ishida

**Affiliations:** Department of Engineering Science, The University of Electro-Communications, Chofu, Tokyo 182-8585, Japan; kyoden@ttf.pc.uec.ac.jp

**Keywords:** aminoxyl, spin transition, exchange interaction, magneto-structural relation

## Abstract

Complex formation of nickel(II) tetrafluoroborate and *tert*-butyl 5-phenyl-2-pyridyl nitroxide (phpyNO) in the presence of sodium cyanate gave a discrete molecule [Ni(phpyNO)_2_(X)_2_] (X = NCO). The Ni-O-N-C_sp_2 torsion angles were reduced on heating; 33.5(5)° and 36.2(4)° at 100 K vs. 25.7(10)° and 32.3(11)° at 400 K. The magnetic behavior was almost diamagnetic below ca. 100 K, and the *χ*_m_*T* value reached 1.04 cm^3^ K mol^−1^ at 400 K. An analysis using the van’t Hoff equation indicates a possible spin transition at *T*_1/2_ >> 400 K. Density functional theory calculation shows that the singlet-quintet energy gap decreases as the structural change from 100 to 400 K. The geometry optimization results suggest that the diamagnetic state has the Ni-O-N-C_sp_2 torsion angles of 32.7° while the *S*_total_ = 2 state has those of 11.9°. The latter could not be experimentally observed even at 400 K. After overviewing the results on the known X = Br, Cl, and NCS derivatives, the magnetic behavior is described in a common phase diagram. The Br and Cl compounds undergo the energy level crossing of the high-/low-spin states, but the NCS and NCO compounds do not in a conventional experimental temperature range. The spin transition mechanism in this series involves the exchange coupling switch between ferro- and antiferromagnetic interactions, corresponding to the high- and low-spin phases, respectively.

## 1. Introduction

Coordination compounds provide us a great opportunity for the development of spin-transition materials [1,2,3,4,5,6,7,8]. Magnetic switches are needed for applications in display, memory, sensor, and related smart devices. Iron(II) (3d^6^)-based spin-crossover (SCO) materials are the most intensively studied [9,10,11,12,13,14], because magnetic and chromic changes would be drastic between the low-spin (LS) *S* = 0 diamagnetic and high-spin (HS) *S* = 2 paramagnetic states. Furthermore, much attention has been paid to SCO near room temperature required for applications under ambient conditions.

In the context of frontier orbital engineering, heterospin systems have an advantage in the wide diversity of paramagnetic centers and practically infinite combinations and mutual geometries. Organic radical species can also be applied to spin sources [6,7,8], and ligands can serve not only as a cap, bridge, or polyhedron regulator, but also as a spin carrier. According to the “metal–radical approach” proposed by Gatteschi et al. [15,16], direct metal-radical coordination bonds afford considerable magnetic exchange interaction. A number of ligands based on 2-azaaryl *tert*-butyl nitroxides (aminoxyls) have been explored for 2p-3d [17,18,19,20,21,22,23] as well as 2p-4f heterospin systems [24,25,26,27,28]. The exchange coupling in radical-coordinated copper(II) (3d^9^) and nickel(II) (3d^8^) complexes is the best understood in the 2p-3d family [15,16,17,18,19,20,29]. Strictly orthogonal arrangement of magnetic orbitals, metal dσ and radical π*, favors ferromagnetic coupling. In contrast, severe angular deformation around the coordination bond gives rise to an overlap between magnetic orbitals and accordingly antiferromagnetic interaction. We have proposed the M-O-N-C_sp_2 torsion angle |*φ*| as a handy metric to evaluate the orthogonal or overlapped dσ and radical π* orbitals [19,24].

**Scheme 1:**
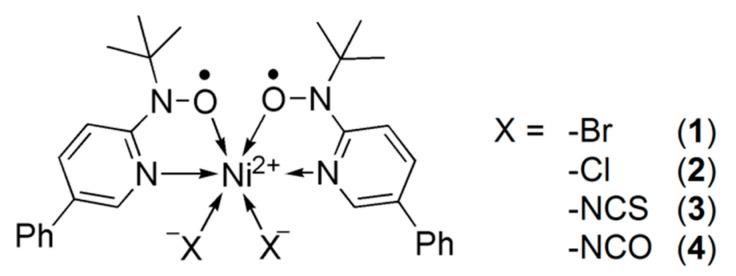
Molecular formula of **1**–**4**.

While exotic spin-transition/SCO systems are explored extensively [6,30], we are developing the *S* = 0 ⇄ 2 spin-transition system other than iron(II) complexes. Three examples have so far been known on the nickel(II)-bis(nitroxide) spin transition between *S*_total_ = 0 ⇄ 2. The reports on [Ni(phpyNO)_2_(X)_2_] with X = Br (**1**, Scheme 1 for the molecular structure) [31], Cl (**2**) [32], and NCS (**3**) [33] afforded a clue to a molecular design, where phpyNO stands for *tert*-butyl 5-phenyl-2-pyridyl nitroxide. A steric effect from anionic co-ligands may tune spin-transition temperature. Here, we will report the results of the X = NCO derivative (**4**).

## 2. Results

### 2.1. Structural Study

Complex [Ni(phpyNO)_2_(NCO)_2_] (**4**) was synthesized by combining solutions of phpyNO and Ni(BF_4_)_2_ in the presence of NaNCO. The elemental, spectroscopic, and crystallographic analyses revealed the absence of any solvent molecules.

**Figure 1:**
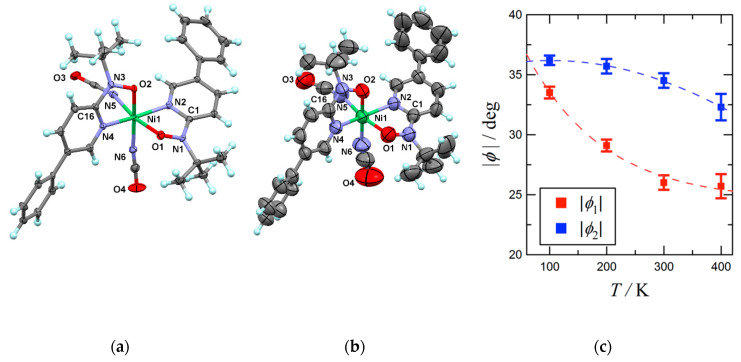
(**a**) X-ray crystal structure of **4** measured at 100 K and (**b**) 400 K. Thermal ellipsoids are drawn at the 50% probability level. Selected atomic numbering is also shown. (**c**) Temperature dependences of torsion angles (|*φ*|) for **4**. Broken lines are drawn for a guide to the eye.

Single-crystal X-ray diffraction measurements on **4** were performed at 100, 200, 300, and 400 K, and Figure 1a,b show the determined structures at 100 and 400 K, respectively. The space group is orthorhombic *Pna*2_1_, and there is a unique molecule in an asymmetric unit. Although the molecular structure of highly related analogue **3** is quite similar, the space group is different [33]. It is probably because the intermolecular interaction regarding the thiocyanate sulfur atoms [34] plays a role of driving force in crystallization of **3**.

A 2p-3d-2p triad was successfully constructed as a discrete molecule of **4**. The co-ligand NCO^–^ is coordinated at a nitrogen atom. Each five-membered chelate ring involves a direct metal-radical bond. The two nitroxide oxygen atoms are located in a *cis* relation.

At 100 K (Figure 1a), the N1-O1 and N2-O3 bond lengths of 1.297(5) and 1.292(5) Å are typical of phpyNO complexes [19]. The Ni-O and Ni-N bond lengths vary in 1.985(4)–2.090(4) Å. The octahedral coordination geometry of the nickel(II) ion indicates *S*_Ni_2^+^ = 1. The bond lengths and bond angles are almost unchanged on heating from 100 K to 400 K. The coordination polyhedron was quantified with the SHAPE program [35]. In fact, the SHAPE measures to the ideal octahedron were 0.887, 0.867, 0.887, and 0.869 for the structures at 100, 200, 300, and 400 K, respectively. The crystal field practically does not change in the entire temperature range. This feature is characteristic of the present transition materials (see below), in sharp contrast to the observation in conventional one-centered SCO materials.

On the other hand, the Ni1-O1-N1-C1 and Ni1-O2-N3-C16 torsion angles were reduced; *φ*_1_ = 33.5(5)° and *φ*_2_ = 36.2(4)° at 100 K vs. 25.7(10) and 32.3(11)° at 400 K (Figure 1b). As Figure 1c displays, *φ* monotonically decreases on heating. The change width is relatively narrow, in comparison with those of **1** and **2** [31,32].

### 2.2. Magnetic Study

**Figure 2:**
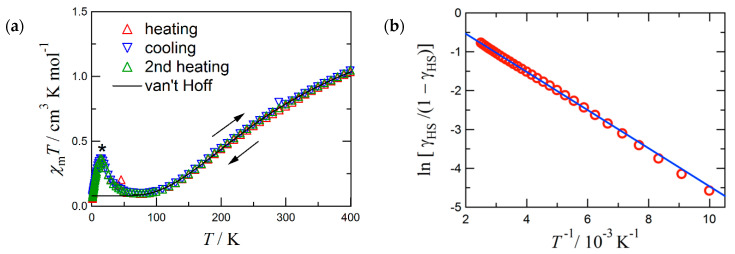
(**a**) The *χ*_m_*T* vs. *T* plot for **4**. An asterisk indicates an impurity signal. (**b**) van’t Hoff plot of the first heating data above 100 K. The solid lines are calculated with the van’t Hoff equation and optimized parameters (see the text). In (**b**) the intercept is ∆*S*/*R* = 0.447, and the slope is −∆*H*/*R* = −492 K, where *R* is the gas constant.

Figure 2a displays the magnetic susceptibility result on **4**. The *χ*_m_*T* value was 1.04 cm^3^ K mol^−1^ at 400 K, being still smaller than 1.75 cm^3^ K mol^−1^ as the spin-only value from one triplet and two doublet species. Furthermore, a positive slope remained at 400 K. On cooling, the *χ*_m_*T* value decreased and approached an almost diamagnetic level. These findings indicate that the presence of considerable antiferromagnetic interaction. According to Okazawa’s formalism [36], the critical torsion angle *φ*_c_ is 21(1)°, and any *φ* values obtained here fell in the antiferromagnetic region. The magneto-structure relationship [19,20,31,36] holds well for the present case.

An impurity peak appeared around 15 K, but the impurity was experimentally inseparable from the specimen (Figure 2a). Probably it would be ascribable to layered or networked magnets based on nickel(II) hydroxides [37,38,39]. Fortunately, this signal occurs only in the lowest temperature region below ca. 50 K, while the *χ*_m_*T* onset ascribable to **4** emerges above ca. 100 K. The impurity signal hardly disturbs the magnetic analysis.

The magnetic data were analyzed quite similarly to that of **3** [33]. The structural study clarifies that the coordination structure is temperature dependent. The van Vleck analysis using an exchange constant is inappropriate. We found that the magnetic data of **4** obeyed the van’t Hoff law (Equation (1)) (Figure 2b). The van’t Hoff analysis is a common method to quantify SCO behavior in the absence of a cooperative effect [40,41].

(1) $$\gamma_{\mathrm{HS}}=\frac{1}{1+\exp[(\Delta H/R)(1/T-1/T_{1/2})]}$$

(2) $$\chi_{m}T=C_{0}\;\gamma_{\mathrm{HS}}+C_{1}$$

The experimental *χ*_m_*T* value was converted to *γ*_HS_, the molar HS fraction (Equation (2)). Parameters *C*_0_ implies the Curie constants for the HS state, and *C*_1_ that of the paramagnetic impurity. The optimized parameters are: *C*_0_ = 3.0(3) cm^3^ K mol^−1^, *C*_1_ = 0.080(3) cm^3^ K mol^−1^, *T*_1/2_ = 1100(300) K, the enthalpy change ∆*H* = 4.1(1) kJ mol^−1^, and the entropy change ∆*S* = 3.7(19) J K^−1^ mol^−1^. The fit curves reproduced the data well. The *C*_0_ value is compatible with the spin-only value expected for the HS state (*S* = 2; 3.0 cm^3^ K mol^–1^). The relatively small *C*_1_ supports the sample purity. The large statistic error of *T*_1/2_ is unavoidable because of only the onset *χ*_m_*T* data available. Although the *C*_0_ parameter seems to be somewhat underestimated and *T*_1/2_ overestimated, owing to a depopulation effect, **4** would display a possible thermal spin transition at *T*_1/2_ >> 400 K.

### 2.3. DFT Calculation Study

**Figure 3:**
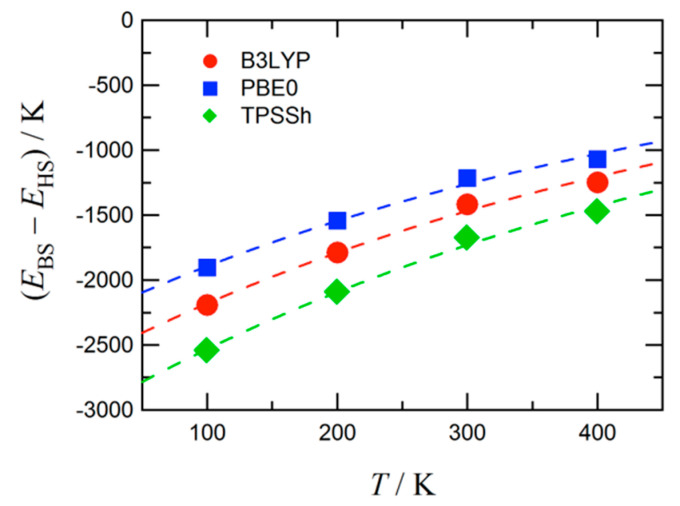
The energy gap between the broken symmetry singlet state (*E*_BS_) and quintet state (*E*_HS_). The functional dependence is also shown. Broken lines are drawn for a guide to the eye.

The atomic coordinates of **4** experimentally determined at 100, 200, 300, and 400 K were subjected to density functional theory (DFT) calculation [42]. The self-consistent field (SCF) energies were computed using the unrestricted DFT theories and 6-311+G(2d,p) basis set. We chose B3LYP, PBE0, and TPSSh [43,44] to check the functional dependence. Figure 3 shows the singlet-quintet energy gaps as a function of temperature. The ground state is singlet at any temperatures, and the gap tends to monotonically decrease, apparently toward null, with an increase of temperature. The PBE0 calculation underestimates the magnitude of the gap, while the TPSSh one overestimates, with respect to the B3LYP result. The three theories qualitatively gave the identical trend.

**Figure 4:**
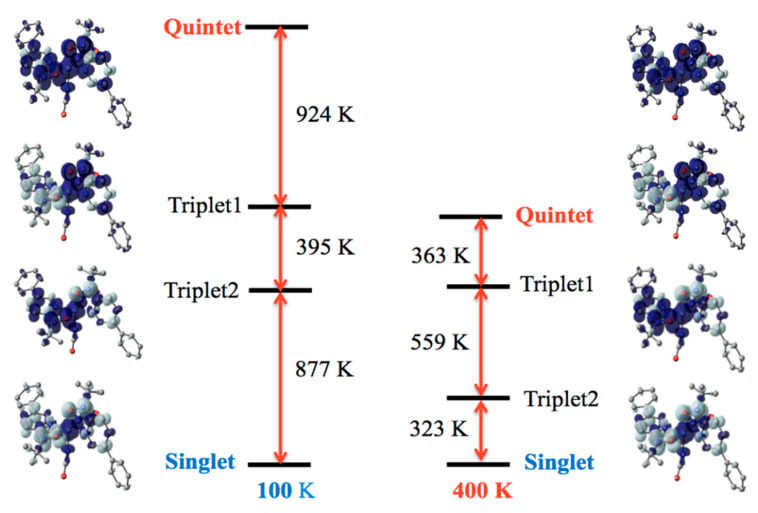
Diagram of the energy levels of the singlet, two triplet, and quintet states for the 100 K and 400 K forms of **4**. Molecules are shown with a spin density map superposed. H atoms are omitted. The positive (dark) and negative spin densities (light) are drawn with an isocontour of 0.002 e^−^ Å^−3^.

Detailed energy level structures were computed with the B3LYP theory, and Figure 4 shows the results of the 100 and 400 K structures of **4**. For the 100 K form (Figure 4, left), the lowest state was singlet, depicted as ↓-↑↑-↓ along the radical-Ni-radical array. The highest state was quintet, ↑-↑↑-↑. The singlet-quintet gap was –2196 K. Two triplet states, ↑-↑↑-↓ and ↓-↑↑-↑, intervene them. The exchange couplings are antiferromagnetic on both sides. For the 400 K form (Figure 4, right), the ground state was also singlet, and the dominant antiferromagnetic behavior retained. However, the most important point is a weakened antiferromagnetic interaction. The singlet-quintet gap was reduced to −1245 K (57% of the corresponding value of the 100 K form). The energy gap to the first excited triplet state became narrow as −323 K (37% of that of the 100 K form). The thermal energy at the temperatures of the magnetic experiments is comparable to this gap. These calculation results are entirely consistent with the magnetic study results.

**Figure 5:**
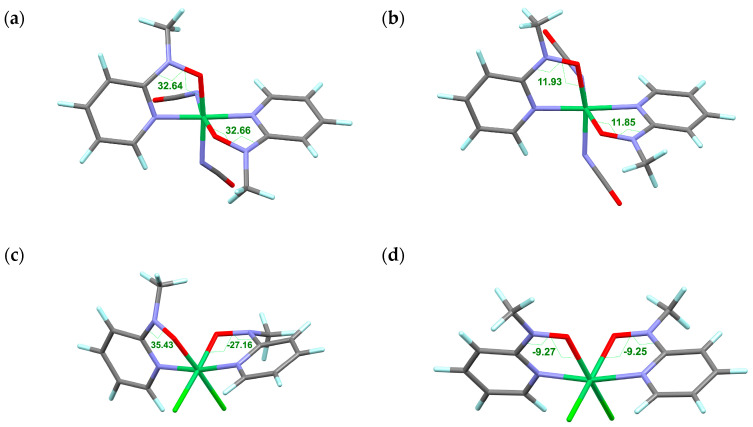
Optimized structures of model 2p-3d-2p compounds: [NiL_2_(NCO)_2_] in the diamagnetic state (**a**) and the *S*_total_ = 2 state (**b**) and [NiL_2_Cl_2_] in the diamagnetic state (**c**) and the *S*_total_ = 2 state (**d**), where L stands for methyl 2-pyridyl nitroxide. The Ni-O-N-C_sp_2 torsion angles are indicated. Color codes: green, Ni; black, C; turquoise, H; blue, N; red, O.

A definitive support for the structure-related exchange coupling switch was offered from geometry optimization results. The optimization was carried out with the B3LYP protocol on the 6-311+G(2d,p) basis set for Ni and 6-31+G(d) basis set for other atoms. To reduce calculation cost, the peripheral *tert*-butyl and phenyl groups were replaced with methyl and hydrogen groups, respectively; namely methyl 2-pyridyl nitroxide (abbreviated as L) was applied as a model ligand. The optimized structures of [NiL_2_(NCO)_2_] are displayed in Figure 5a,b. The Ni-O-N-C_sp_2 torsion angles of the diamagnetic state are 32.7°, whereas those of the *S*_total_ = 2 state are 11.9°. They possess a two-fold symmetry. Although the conformation of the NCO groups varied during the energy minimization, it is safely concluded that intramolecular 2p-3d antiferromagnetic coupling favors a large torsion, while ferromagnetic coupling a small torsion. The calculation well reproduced the experimental values of the diamagnetic phase. However, the value of 11.9° could not be experimentally observed even at 400 K. In other words, the thermal energy of 400 K is insufficient to cause spin transition.

To remove the conformational freedom in the co-ligands, another calculation was performed on a model compound [NiL_2_Cl_2_]. The geometries were optimized for the diamagnetic and *S*_total_ = 2 states on the same calculation level (Figure 5c,d, respectively). The former has *φ* = 35.4 and −27.2°, while the latter has *φ* = −9.3°. The experimental values of **2** are 38.9(2) and −29.2(2) at 85 K and 25.8(3) and 13.0(4) at 400 K [32]. In that case, the 85 K and 400 K structures are concluded to belong to the LS and HS phases, respectively. The calculation well reproduced the experimental values for the diamagnetic phase in particular. The sign of *φ* means a chirality of the torsional direction. The absolute values are important for the orbital overlap, but it must be pointed out that the same and different signs indicate “conrotatory” and “disrotatory” twisting directions, respectively, in a molecule. The DFT calculation reproduced as well such observations in the diamagnetic state.

## 3. Discussion

The nickel(II)-radical exchange coupling parameter varies in a wide range, actually 2*J*/*k*_B_ = +409 K [19] to −1400 K [45], and is very sensitive to the overlap between the nickel(II) and radical magnetic orbitals. Thanks to this, the unique spin transition materials are realized. The novelty of the present spin transition mechanism resides in the ferro- and antiferromangetic exchange coupling switch, corresponding to the HS and LS phases, respectively.

Now let us move to discuss: What is the driving force of the torsional deformation found in **4**? Why both wings synchronously move?

**Figure 6:**
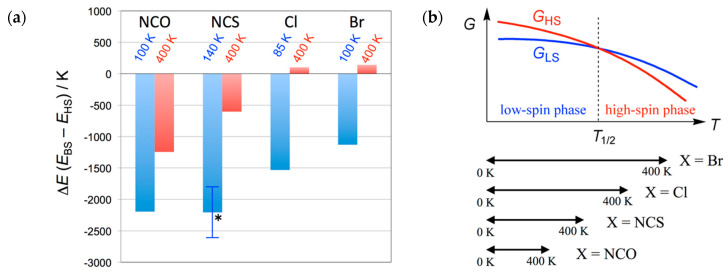
(**a**) Temperature dependence of the energy gap between the broken-symmetry singlet state (BS) and quintet state (HS) on the determined structures of **4** (X = NCO), **3** (NCS), **2** (Cl), **1** (Br). The temperatures of structure determination are noted. An asterisk indicates an averaged value of the two independent molecules. For comparison on the same level, the data of **2** were updated as ∆*E*/*k*_B_ = −1533 and +102 K at *T* = 85 and 400 K, respectively. (**b**) A schematic Gibbs energy diagram together with the experimental temperature windows for spin-transition materials **1**–**4**.

Figure 6a summarizes the calculation results on **1**–**4** [31,32,33]. In sharp contrast to the results on **3** and **4**, the spin multiplicity of **1** and **2** was calculated to be HS at 400 K with the singlet-quintet energy gaps were +141 and +102 K for **1** and **2**, respectively. They are spin-transition materials due to the exchange coupling switch. On the other hand, the thermal energy is insufficient for spin transition even at 400 K for **3** and **4**. Overall, the four compounds are documented in a common phase diagram, as illustrated in Figure 6b. The critical temperature *T*_1/2_ is defined by the level crossing point. Compounds **3** and **4** exhibited an indication of a possible spin-transition, as a result of *T*_1/2_ outside of the temperature window of conventional magnetic measurements. Even if the temperature is elevated above *T*_1/2_, the magnetic susceptibility did not reach the HS limit, but approached the paramagnetic limit, owing to depopulation from the HS state.

We have to state briefly the *T*_1/2_ order: **1** < **2** < **3** < **4**. This chemical trend can be explained in terms of the steric effects from the co-ligands [33]. The order of the van der Waals radii is *r*(N) < *r*(Cl) < *r*(Br) [46], and the ligating atom mainly affects the steric congestion in the first coordination sphere. The terminal sulfur and oxygen atoms may play an auxiliary role. Small co-ligands can accommodate out-of-plane displacement of adjacent ligating atoms (i.e., nitroxide oxygen atoms), giving rise to stabilization of the LS state and accordingly a high-temperature shift of *T*_1/2_.

All the compounds exhibited a single-crystal-to-single-crystal transition, as often found in organic-based spin-transition materials [47]. The enthalpy change supplying the atomic dislocation energy is regulated by the entropy change (*T*_1/2_∆_tr_*S* = ∆_tr_*H*), which is generally small. The entropy due to the vibrational contribution is negligible because the structural change is merely the torsional deformation in **1**–**4**, in comparison with those of the bond length changes in the conventional one-centered SCO compounds like iron(II) complexes. Consequently, the spin entropy change suffices the enthalpy change of the spin transition accompanied by the small structural transition. In short, the spin transition of the present series seems to be entropy-driven [48,49]. The Ni-O-N-C_sp_2 angular deformations in **1**–**4** are always synchronized on both wings, and every interaction tends to alter from ferro- to antiferromagnetic on lowering temperature, because the spin multiplicity could be minimized (in this case, *S*_total_ = 0). This notion also holds for the result on a spin-transition copper(II)-bis(nitroxide) analogue, [Cu(phpyNO)_2_(H_2_O)_2_](BF_4_)_2_ [50]. In that case, no synchronous transition occurs between the *S*_total_ = 3/2 and 1/2 states. Only one side deformation is enough for minimization of *S*_total_.

There have been several reports on spin-transition phenomena in copper(II)-radical systems. Okazawa et al. reported the spin transition of Cu^2+^-nitroxide compounds, and the mechanism is quite similar to the present Ni^2+^ case [50,51]. Namely, the Cu-O-N-C_sp_2 angular deformation causes the exchange coupling switch. On the other hand, Ovcharenko et al. reported the spin-transition Cu^2+^-nitroxide compounds [52,53], and the mechanism involves a switch of the role of axial and equatorial coordinations [54]. Rey et al. reported another type of the spin-transition Cu^2+^-nitroxide materials [55], where the coordination environment is changed between a square pyramid and a trigonal bipyramid. In contrast, our group focused on octahedral nickel(II) complexes. A coupling switch takes place at any coordination sites, axial or equatorial. Rey et al. named a “pseudo-spin-transition” material that shows a switch between strong and weak ferromagnetic couplings. Thus, this article is supposed to report another example of a “pseudo-spin-transition” material that shows a switch between strong and moderate antiferromagnetic couplings.

## 4. Materials and Methods

The following procedure of the preparation of **4** is typical. An acetone solution (1.0 mL) containing phpyNO [19] (48.4 mg; 0.201 mmol) was combined with a water (0.60 mL) − methanol (0.40 mL) mixed solution containing Ni(BF_4_)_2_·6H_2_O (35.0 mg; 0.103 mmol) and NaNCO (13.8 mg; 0.212 mmol) at room temperature. After two days black rectangle-shaped crystals of **4** were formed, which were collected on a filter, washed, and dried. The yield was 36.3 mg (0.0581 mmol; 58%). Mp. 196 °C (dec.). IR (neat, attenuated total reflection) 3065, 2985, 2195, 1454, 1313, 1258, 1182, 1012, 764, 692, 610 cm^−1^. Anal. Calcd. for C_32_H_34_N_6_NiO_4_: C, 61.46; H, 5.48; N, 13.44%. Found: C, 61.63; H, 5.64; N, 13.52%.

X-ray diffraction data of a single crystal of **4** were recorded on a Rigaku Saturn70 CCD diffractometer using graphite monochromatized Mo Kα radiation (*λ* = 0.71073 Å). The structures were solved in the CRYSTALSTRUCTURE application [56], and the parameters were refined in the SHELXL module [57]. Hydrogen atoms were located at calculated positions. Important crystal data are listed in Table 1. CCDC numbers 2018891 and 2018892 for the structures at 100 and 400 K, respectively. 

Magnetic susceptibilities of polycrystalline **4** were acquired on a Quantum Design MPMS XL-7 SQUID magnetometer (San Diego, CA, USA). Temperature was scanned from 1.8 to 400 K at a constant magnetic field of 0.5 T. The diamagnetic susceptibility was calculated from Pascal’s constant [58].

Density functional theory (DFT) calculations were carried out on the Gaussian16 Revision C.01 [59]. The broken symmetry method [42] was applied. For analyzing energy level structures of **4**, the self-consistent field energies were calculated with the experimentally-determined coordinates. For geometrical optimization, a tight conversion was applied from the experimental coordinates as a starting structure.

## 5. Conclusions

When the singlet-quintet energy gap is relatively small, there is a chance of spin transition. After overviewing the results on [Ni(phpyNO)_2_(X)_2_] (L = Br, Cl, NCS, NCO), the magnetic behavior is described in a common phase diagram. The Br and Cl compounds undergo the energy level crossing of the HS/LS states and behave as a spin-transition material. On the other hand, the NCS and NCO compounds do not in a conventional experimental temperature range. The HS/LS phases originate directly from the intramolecular 2p-3d ferro-/antiferromagnetic exchange couplings.

The present solid-state/solid-state structural transition, or more likely crossover, involves a very slight torsional deformation around the coordination bond. Thus, the preset scenario can be regarded as a novel mechanism operative in multi-centered SCO. The spin state is regulated by antiferro-/ferromagnetic balance in multi-centered systems, in place of the aufbau/Hund balance in one-centered systems. The aufbau principle works in gapped d-orbitals caused by a ligand field as well as in gapped bonding and antibonding molecular orbitals caused by interaction between magnetic orbitals.

Finally, we have to emphasize that *J* is a variable of *T* in the analysis of the magnetic data. The single-point X-ray crystal structure analysis cannot reveal a hidden structural transition, but combining detailed works from structural chemistry and magnetochemistry can solve such problems.

## Figures and Tables

**Table 1:** Selected crystallographic parameters of **4**.

Compound	[Ni(phpyNO)_2_(NCO)_2_] (4)	
*T/*K	100	400
Formula, formula weight	C_32_H_34_N_6_NiO_4_, 625.36	
Crystal system	orthorhombic	
Space group	*Pna*2_1_	
*a/*Å	19.489(4)	19.727(9)
*b/*Å	8.992(2)	9.409(4)
*c/*Å	17.316(4)	17.217(7)
*V/*Å^3^	3034.5(11)	3196(2)
*Z*	4	4
*d*_calcd_/g·cm^−3^	1.369	1.300
*μ* (MoKα)/mm^−1^	0.686	0.652
No. of unique reflections	6948	7227
*R*(*F*) (*I* > 2σ(*I*)) ^a^	0.0525	0.0805
*wR*(*F*^2^) (all reflections) ^b^	0.0989	0.1932
Goodness-of-fit parameter	1.054	1.155

^a^*R* = Σ[|*F*_o_| − |*F*_c_|]/Σ|*F*_o_|; ^b^ *wR* = [Σ*w*(*F*_o_^2^ − *F*_c_^2^)/Σ*wF*_o_^4^]^1/2^.
